# Development of a Novel Coaching Platform to Improve Tackle Technique in Youth Rugby Players: A Proof of Concept

**DOI:** 10.3390/s22093315

**Published:** 2022-04-26

**Authors:** Ed Daly, Patrick Esser, Alan Griffin, Damien Costello, Justin Servis, David Gallagher, Lisa Ryan

**Affiliations:** 1 Department of Sport, Exercise & Nutrition, Atlantic Technological University (ATU), H91 T8NW Galway, Ireland; ed.daly@gmit.ie (E.D.); alan.griffin@gmit.ie (A.G.); damien.costello@gmit.ie (D.C.); jservis@gmail.com (J.S.); dgallagher1111@gmail.com (D.G.); 2 Department of Sport, Health Sciences and Social Work, Oxford Brookes University, Oxford OX3 0BP, UK; pesser@brookes.ac.uk

**Keywords:** injury risk, tackle safety, inertial measurement unit, rugby union

## Abstract

Rugby union is a field sport that is played at amateur and professional levels by male and female players globally. One of the most prevalent injury risks associated with the sport involves tackle collisions with opposition players. This suggests that a targeted injury reduction strategy could focus on the tackle area in the game. In amateur rugby union, injuries to the head, face and shoulder are the most common injury sites in youth rugby playing populations. A suboptimal tackle technique may contribute to an increased injury risk in these populations. One proposed mitigation strategy to reduce tackle-related injuries in youth populations may be to increase tackle proficiency by coaching an effective tackle technique. The present study aimed to demonstrate a proof of concept for a tackle technique coaching platform using inertial measurement units (IMUs) and a bespoke mobile application developed for a mobile device (i.e., a mobile phone). The test battery provided a proof of concept for the primary objective of modelling the motion of a player in a tackle event. The prototype (bespoke mobile application) modelled the IMU in a 3D space and demonstrated the orientation during a tackle event. The participants simulated ten tackle events that were ten degrees above and ten degrees below the zero degree of approach, and these (unsafe tackles) were indicated by a red light on the mobile display unit. The parameters of ten degrees above and below the zero angle of approach were measured using an inclinometer mobile application. These tackle event simulations provided a real-time stream of data that displayed the angle of tackles on a mobile device. The novel coaching platform could therefore constitute part of an injury reduction strategy for amateur or novice coaches to instruct safer tackle practice in youth rugby playing populations.

## 1. Introduction

Rugby union is a field sport that is played at amateur and professional levels by male and female players globally. The most recent data from World Rugby report that the number of registered players stands at approximately 9.6 million across 123 countries [1]. One of the most prevalent injury risks associated with the sport involves tackle collisions with opposition players. Injury data from rugby surveillance projects in Ireland involving amateur rugby players (Irish Rugby Injury Surveillance (IRIS), 2018–2019) cited that the most common injuries occur in the physical contact areas (i.e., tackling, rucks) of the game [2]. In this report, it was recorded that tackling an opponent accounted for the majority of match and training injuries in amateur rugby (59% of the overall injury burden). IRIS also found that the most frequently reported match injury for both male and female players was concussion (11% and 19%, respectively). In the United Kingdom, injuries to the head, face and shoulder were the most common injury sites in amateur youth rugby playing populations, with over 50% of the injuries occurring in the tackle or collision areas of the game [3]. Data provided by research in school-level rugby union in Australia, with a similar age profile (age 13 ± 4.5 y), suggested that injuries to the head and face accounted for 33.7% (n = 112) of all injuries [4]. From the overall total of 332 injuries, there were 61 cases of suspected concussion injuries. Additional research found that amateur players who had an incorrect head position relative to the ball carrier resulted in a higher incidence of concussions, neck injuries and facial fractures when compared with players that performed tackles with the correct head position (the injury incidence with incorrect head positioning was 69.4/1000 tackles, compared with the injury incidence with correct head positioning of 2.7/1000 tackles) [5].

When compared with professional rugby, the injury incidence involving tackling in adult amateur rugby was lower than that in the professional game but had a higher incidence rate when compared with youth rugby [6]. This suggests that future injury reduction strategies must be aimed towards the tackle area in the game of amateur rugby union [6]. In youth rugby, a suboptimal tackle technique may contribute to an increased injury risk, but this risk can be reduced by implementing safer techniques during training sessions [7]. At youth rugby levels, it has been reported that many coaches have relied on informal or anecdotal resources (e.g., previous playing experience or observation of matches) to develop their knowledge of tackle training [8]. 

One proposed mitigation strategy to reduce tackle-related injuries in youth populations may be to enhance coaching skills that increase tackle proficiency and technical capacity in youth rugby players [8,9]. Additional research supports current rugby coaching practice, whereby a player should have their head positioned upwards and be facing the ball carrier to improve tackle technique proficiency [10]. This training technique results in a ‘head up’ position which allows the tackler to perform a shoulder tackle on the mid torso of the opposing player. This suggested tackle technique can assist the head placement of the tackler once the initial contact is instigated and can result in a more proficient tackle technique [10]. 

Evidence suggests that tackle mechanism-related research may act as a mitigating factor in reducing tackle-related injuries [11]. Further research has proposed that the tackler trunk had a direct effect on head kinematics when utilising tackles aimed at the mid and/or lower trunk of the player being tackled [12]. With respect to youth rugby, it has been advised that the ‘head up and forward before contact’ coaching cue may result in safer contact techniques [13]. This study found that players who were deemed to be concussed displayed a ‘down’ or ‘away’ head position on contact which may lead to an in-creased risk of head or neck injuries [13]. This coaching instruction is a technique that could be incorporated by coaches to instruct players to maintain the ‘up and forward’ head position into tackle contact and not only in anticipation of contact with an opposing player. Injury risk may be reduced when coaches are able to observe and measure tackle proficiency by quantifying the quality of the tackle technique. 

For example, research has demonstrated the use of observable technical criteria which outline a list of clear distinct phases of tackling (i.e., pre-contact, contact and post-contact). This study analysed the tackle technique of each player which was scored one point or zero depending on the action performed during pre-contact, contact and post-contact [14]. For example, during the pre-contact phase, if the tackler was in a ‘head up and forward’ angle of approach, this was deemed as a good action and awarded one point. The sum of the points allocated was used to determine the technical proficiency score of the player during tackling or contact events. Further research has found that tacklers that had an incorrect head position or that had intentionally used a poor technique tended to result in more injuries as the players’ head and neck were in an incorrect position [5]. 

By extension, these safer tackle techniques (i.e., ‘head up and forward’ and zero angle of approach) could be applied to other contact areas (e.g., rucks, scrums) to mitigate against injury in contact areas of the game. In amateur youth rugby, coaches that deliver tackle training may have knowledge gaps specifically related to their own competencies in coaching an effective tackle technique [9]. If coaches were to implement a diagnostic prescriptive model to address areas of improvement and monitor ‘ideal’ movement in all contact areas, this may reduce injury risk as coaches could instruct the optimal movement technique during contact training [15]. By utilising contemporary wearable technologies such as inertial measurement units (IMUs), the movement of players can be captured. The use of IMUs is well established in the literature [16]. There is support for the use of IMUs as tracking devices for human movement in sport and translating specific movement actions which can be detected and extracted using IMUs [17]. A recent study suggested that the use of microprocessor technology (such as that contained in IMUs) is a valid method to detect human motion in contact events in rugby union [18]. At present, IMUs are not linked to visualisation platforms for coaching tackle technique.

The present study aimed to demonstrate a proof of concept for a novel tackle technique training platform using IMUs and a bespoke mobile application developed for a mobile device (e.g., a mobile phone or tablet). It was hypothesised that developing a mobile coaching platform which utilises live IMU data could potentially lead to improved coaching strategies for amateur and novice coaches regarding tackle technique. This coaching platform could, in turn, reduce the incidence of upper head, neck and facial injuries in youth rugby by better informed coaching practice. The platform could therefore potentially constitute part of an injury reduction strategy for amateur or novice coaches that are responsible for instructing safe tackle practice in youth rugby playing populations. 

## 2. Materials and Methods

A novel prototype mobile application was developed by the research team for use on mobile phones. The prototype application was initially developed for laptop usage and further engineered for use on a mobile phone. The development of this mobile functionality (e.g., mobile phone/tablet) was explicitly for use in an applied setting by amateur coaches for training sessions with amateur youth rugby union players. 

### 2.1. Technical Description—Inertial Measurement Units (IMUs)

The Shimmer3 Wireless Sensor Unit (Shimmer Research Ltd., Dublin, Ireland) is a wearable device that contains two accelerometers, a gyroscope and a magnetometer. This combination of sensors is an IMU with nine degrees of freedom (DFs). The proposal would use the IMUs to capture the orientation of a player’s body in a tackle simulation training exercise by using kinematic data which notionally focused on the orientation of a player’s upper body (i.e., scapulae and shoulders).

### 2.2. Technical Description—Development of the Prototype Mobile Application

This bespoke mobile application is a lightweight application connecting the IMU data streams directly to a 3D model in the prototype mobile application. The axial output from the IMU is converted to Euler angles that are applied to the model in real time within the mobile application. Initially developed for a laptop using the Unity 3D Game Engine, it was further engineered and ported to a mobile device to increase access to data during training sessions. Unity3D was used in the development of the Android application as it has an integrated physics engine which was used when manipulating the nine DFs provided by the IMU. Converting from Euler angles to quaternions was necessary to manipulate the data returned from the IMU. 

**Figure 1:**
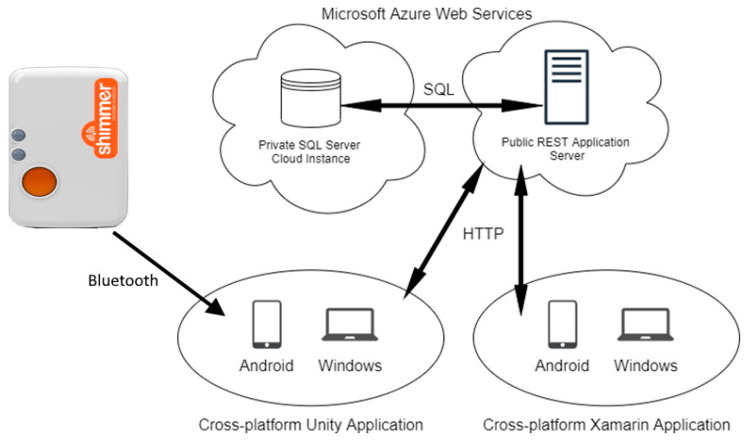
Program architecture and data flow.

The system is composed of three core applications and a database (see Figure 1). There are two cross-platform applications and a representational state transfer application programming interface (REST API) server application backed by the database instance. The applications may be deployed on Android or Windows 10 (Microsoft Corporation, Redmond, WA, USA) devices, and the server application was deployed to Azure Web Services (Microsoft Corporation, USA) for this project.

### 2.3. Ethics

Ethics for this study was granted by the Taught Programme Research Ethics Committee (TPREC) of the Atlantic Technological University (reference number GMIT_TPREC_250120). All participants provided written and/or verbal informed consent prior to data collection.

### 2.4. Data Collection

Four male participants (age 18.1 ± 0.6 y/height 179.5 ± 3.3 cm) were fitted with a GPS vest (VX Sport Systems), and the IMUs were inserted into the GPS pocket located between the scapulae of the GPS vest. This enabled a constant position on the participant to measure the roll, pitch and yaw (i.e., rotation around the XYZ axes). The participants calibrated the IMUs by assuming a standing position and completing rotational, hinge and gait movement patterns.

Prior to the tackle simulation tests, all participants undertook a warmup that included technical instruction (the participants were not rugby players), a physical mobilisation and a familiarisation with each test condition. The participants were instructed to approach a foam-padded surface which simulated the contact area during tackle events to become familiarised with the test area. Once the GPS vests and IMUs were worn by the participants, they were instructed to approach the padded surface with their hips hinged and their heads positioned upward parallel to the ground plane. When the participants touched the padded area with their shoulder, tape (zinc oxide) was applied to the padded area to measure where their shoulder met the padded area; this indicated each participant’s zero angle of approach to the foam-padded surface when testing commenced. 

**Figure 2:**
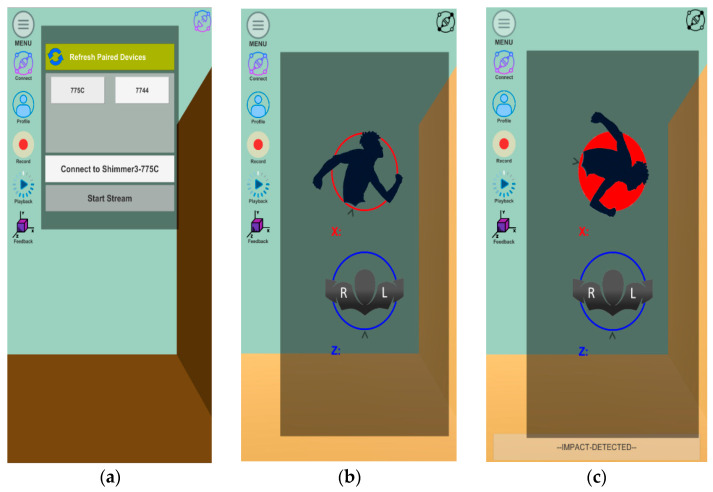
(**a**) Mobile application connecting to the inertial measurement unit (IMU); (**b**) calibrating IMU to the participant; and (**c**) modelling an unsafe tackle indicated by the ‘red light’ display.

Each participant engaged in ten static simulated tackle events (five attempts on the left shoulder and five attempts on the right shoulder). These static tackle simulations were executed in a parallel direction (i.e., simulating a zero angle of approach to the ground plane of the test area). The simulated tackles that were performed in this manner produced a green light on the prototype mobile application which indicated a safe tackle (see Figure 2. The participants simulated ten tackle events that were approximately ten degrees above and ten degrees below these parallel tolerances, and these (unsafe tackles) were indicated by a red light on the mobile display unit (see Figure 2). The parameters of ten degrees above and below the zero angle of approach were measured and marked on the foam-padded surface using an inclinometer mobile application (Clinometer, France).

## 3. Results

**Figure 3:**
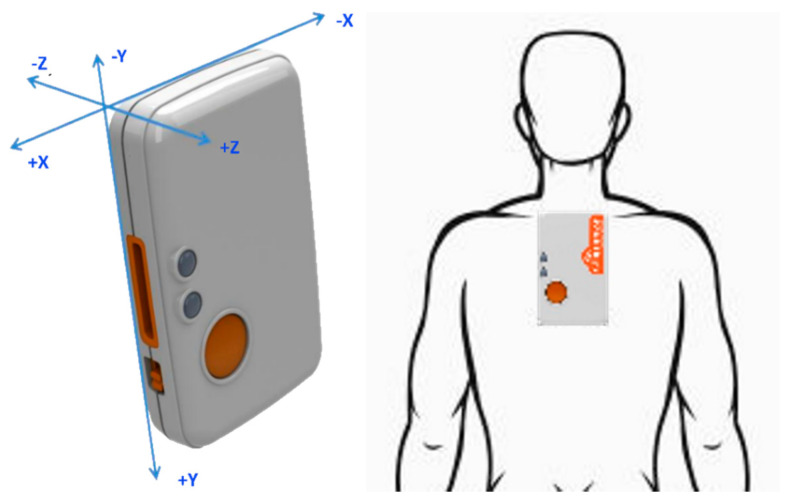
Shimmer axes of rotation demonstrating indicative placement of the IMU.

The test battery provided a proof of concept for the primary objective of modelling the motion of a participant in a tackle event using an IMU and a novel prototype mobile application. The prototype application modelled the IMU unit in a 3D space and demonstrated the orientation during a tackle event to the researchers. These tackle event simulations provided a real-time stream of data that displayed the angle of tackle for the researchers to observe on the mobile phone device. The data collected were saved and used to replay the tackle technique to each participant which assisted the researchers in modifying and coaching the participants’ tackle posture characteristics. The data returned were used to calculate the roll, pitch and yaw of the IMU on the participant and update the 3D model on the mobile phone device. The pitch, roll and yaw are the rotations about the X, Y and Z axes, respectively. These axes are shown in Figure 3 for the IMU which are aligned on the participant with the Y axis along the spine and the X axis across the shoulders.

Table 1 shows a sample of the data returned from the IMU during the testing phase of the project. Four readings are provided with 38 data points for each. The IMUs return data from the accelerometers, the gyroscope and the magnetometer. These data are used by the processor within the IMU to produce the axis angles and the quaternion values. For the purposes of this project, the data from the low-noise accelerometer. gyroscope, magnetometer, axis angle and quaternion were saved and imported for use in displaying the orientation of the Unity 3D model. Each data point is timestamped, and this is used to order the data points during a replay of a tackle event.

**Figure 4:**
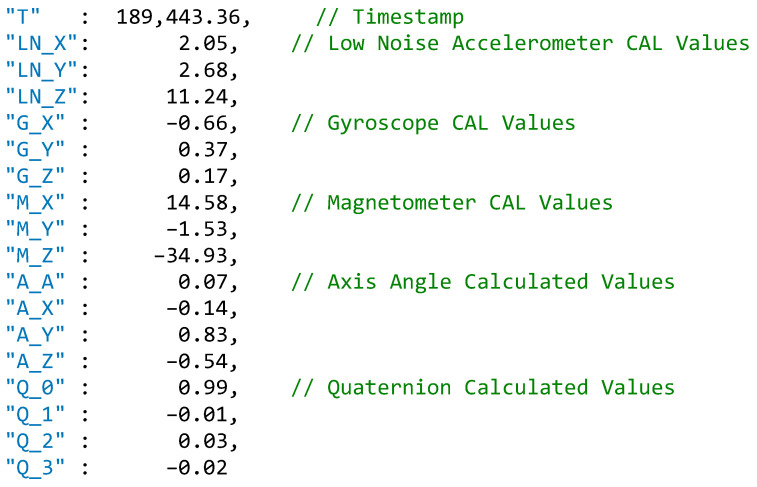
JavaScript Object Notation (JSON) formatted data for a single reading.

Based on these data, the axis angle and the quaternion values are calculated by the IMU processor during the data streaming process. These values are filtered for use by the Unity application and stored in a JavaScript Object Notation (JSON) format for ease of processing within the application. These data are illustrated in Figure 4 for a single reading.

**Figure 5:**
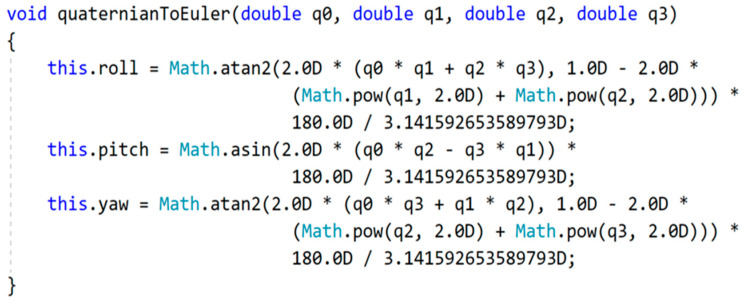
Code sample for calculating the roll, pitch and yaw.

The quaternions are used to calculate the roll, pitch and yaw to be reflected in the Unity 3D model. The calculations for these are conducted with the method shown in the code sample in Figure 5 and are based on standard math formulas for this purpose.

Once these values are calculated, the 3D Unity application updates the orientation of the model and demonstrates the modelling of a player’s motion, using the IMU in a tackle event in Figure 2. Tackle angles that were deemed safe (i.e., within ten degrees of a zero angle of approach) displayed a ‘green’ light on the mobile display, and tackles that were deemed unsafe displayed a ‘red’ light (i.e., outside the safety parameters of ten degrees above or below the zero angle of approach) on the mobile phone display (see Figure 2).

## 4. Discussion

The objective of this study was to demonstrate a proof of concept for a novel tackle technique training platform using IMUs and a bespoke mobile application developed for a mobile device (e.g., a mobile phone or tablet). This novel mobile coaching platform could be used to capture the motion and orientation of a participant’s body during tackle events in rugby. The prototype coaching platform utilised a novel mobile phone application and IMUs as a sensor-based recording system. This study successfully provided a proof of concept for the coaching platform to demonstrate safe or unsafe angles of approach during tackle events in tackle technique training for amateur coaches. Participants demonstrated tackle angles that provided kinematic data of the upper body in static simulated tackle technique training. As this is a prototype tackle technique coaching platform, further development remains to refine the visual warning system for amateur coaches. The current prototype mobile phone application can provide visual feedback to the coach using a traffic light system (green or red) based on the angles of rotation around the XYZ axes. In this study, suggested tolerance parameters (i.e., ±10° the zero angle of approach) of the tackle were based on existing kinematic rugby tackle data [12,15]. The system displays a green or red indicator light along the axes depending on the set tolerance parameters utilised in the study. The model on the mobile user interface (UI) changes colour as the angle of rotation approaches the tolerance parameters (ten degrees above or ten degrees below the parallel plane) which indicates either safe (green) or unsafe (red) angles of approach in a tackle event. The suggested parameters (±10°) were primarily selected as a guide to novice coaches, and assist in a ‘head up and forward’ approach to tackle safety. By adopting this approach to coaching safer tackle technique, it may assist in improving tackle posture and enable novice youth players to target the upper legs and lower trunk area of the ball carrier in live tackle scenarios. 

As previously mentioned, coaching appropriate safer tackle technique may reduce injury risk in youth players or in adult amateur players [19]. The data provided by this platform could constitute part of an injury reduction strategy for amateur or novice coaches. Recent research has suggested that when coaches emphasise a proficient tackle technique in training, it has a positive relationship with the perception of importance for players in tackle training and overall injury reduction [20]. The data provided by this novel coaching platform could improve the knowledge of tackle technique and the development of better implementation techniques for the coach. Where coaches are capable of instructing, guiding and offering feedback to players on tackle technique, the players’ tackle proficiency and self-efficacy progress in conjunction with better technique [20]. 

A future development of this platform for amateur coaches would be to validate the novel coaching platform against video analysis of tackle technique as video analysis is a recognised method for analysing biomechanical motion in rugby training or during active competition [21]. Evidence suggests that combining video analysis and wearable technology such as IMUs is a more robust method to investigate human motion in a sports setting [22,23,24,25]. This application of using video analysis for tackle technique may alter lower extremity kinematics for inexperienced youth rugby players which could reduce head accelerations during tackle events [26]. One advantage of using video analysis in sport is the ability to infinitely pause, replay and focus on specific segments of training or competitive conditions. This capability to pause and replay could aid amateur coaches in identifying tackle technique deficits, with a greater awareness of injury risk in rugby settings [27]. 

The present study offers a potentially novel platform to assist amateur coaches in identifying injury risk and improve the safety of tackle technique for youth rugby union players without the use of additional video analysis. The novel platform developed for this study has the capability of instant feedback or review of individual tackle technique during a training session. The platform has the added functionality to store and review tackle training sessions for a number of players which could be reviewed by the player and coach at a later date. The use of this novel coaching platform could facilitate vision training for tackle performance and may assist in developing the anticipation skills required for tackling and contact skills in rugby union. Vision training has been demonstrated to be a proposed concussion reduction strategy as it may assist in improving the ability to ‘scan’ the visual field for opponents and anticipate physical impacts [26]. The use of mobile applications and technology for sports coaching is well established. Technology and mobile applications can provide the means of gathering and analysing data which can then be translated into coaching practice for amateur coaches. 

### Limitations

There are limitations to the current research, even though it has demonstrated that data gathered from wearable sensors (IMUs) can increase the potential to correct tackle technique using a mobile coaching application. The focus of this initial stage of research was to demonstrate that IMUs and a mobile application could model an optimum safe tackle technique in training exercises by suggesting an optimum approach to the development of a safe tackle technique. In turn, this type of technology could assist a novice coach in developing a safer tackle posture for tacklers (i.e., ‘head up and forward’ posture). The current research could be further enhanced to identify specific causes of injury that occur with a poor tackle posture during tackle events in training and ultimately in live match play. As the game of rugby is a dynamic field-based sport, it is challenging to predict how tackles will be executed in real time. Consequently, the modelling in this proof of concept is constrained by limitations in the existing mobile application to adjust for non-optimum tackle angles. In many live tackle situations, the tackler may be unable to execute the optimum tackle angle. During these instances, the tackler may not be able to target the upper legs or lower trunk area of the ball carrier. This may mean that a tackler executes a tackle below the knees or above the waistline of the ball carrier. In these scenarios, these tackle angles would be outside the current parameters of ±10° and deemed unsafe using the current mobile application. Future iterations of the mobile application may need a facility where the optimum tackle angle could be adjusted, as this may replicate a more ‘real’ reflection of tackling in live matches. 

A further limitation of the current research is in respect to the ±10° on tackle approach. The current research selected ±10° based on existing kinematic rugby tackle data which suggested a safe tackle approach would target the upper leg and lower trunk. Using an initial zero angle of approach to the tackle and allowing ±10° tolerance would mimic a would-be tacklers angle of approach to an oncoming ball carrier. The rationale for using ±10° as an angle of approach as that it may assist in the ‘head up and forward’ motion for players to recall, and novice coaches to cue in coaching tackle safety. Equally, it could be suggested that ±15° or other angle variations could be used as a safe tolerance; however, this was beyond the scope of the current paper. Using different tolerances (e.g., ±5°, ±15°, ±20°) may yield different results and assist in furthering the development of the current mobile application. 

Future research development of this proof of concept will be to compare the current platform with video analysis as a means of reliability and validity testing. The current study demonstrated that it is possible to model static tackling posture characteristics in novice rugby players. The second phase will involve modelling live tackling events that measure proficient angles of approach to tackle technique and measurement of the forces during tackle impacts. This development will assist in the overall quality of coaching for tackle technique training in amateur rugby players and amateur coaches. 

A final limitation of the present study was that all participants were male, which meant that the research team was unable to conclude if this prototype coaching platform may be suitable for female rugby players. This should be a notable consideration due to the differences in body mass and muscular distribution between male and female players in the amateur game of rugby union. This may lead to the development of alternate means of tackle technique coaching platforms and tackle proficiency strategies for females due to these parameters.

## 5. Conclusions

The objective of this study was to demonstrate a proof of concept for a novel tackle technique training platform using IMUs and a bespoke mobile application to capture the orientation of a youth rugby player’s body during a tackle event. This study successfully demonstrated the development of a novel cross-platform software application for use in combination with IMUs. Wearable technology combined with the development of bespoke mobile applications has the potential to capture motion and potentially improve tackle technique proficiency to reduce injury risk in youth rugby. With advances in wearable technology, and associated reductions in cost, the development of novel mobile applications for improving tackle technique will enhance the potential for such applications to be widely utilised in amateur and community sport settings. In addition, this use of wearable technology and the developed bespoke mobile application could be utilised to monitor improper body position during activities of daily living, i.e., sitting posture or examining different physical strengths (asymmetries) of the left or right part of the body due to physiological or anatomical changes.

## Figures and Tables

**Table 1:** Sample reading of data returned from the inertial measurement unit (IMU). Accelerometer values are in metres per second squared (m/s^2^), the gyroscope returns degrees per second (°/s) and the magnetometer provides flux (cgs) values.

	Reading 1	Reading 2	Reading 3	Reading 4
Timestamp_RAW	11,461,017.00	11,467,417.00	11,473,817.00	11,480,217.00
Timestamp_CAL	189,443.36	189,638.67	189,833.98	190,029.30
Low_Noise_Accelerometer_X_RAW	2006.00	2006.00	2007.00	2006.00
Low_Noise_Accelerometer_X_CAL	2.05	2.08	2.05	2.07
Low_Noise_Accelerometer_Y_RAW	2064.00	2062.00	2064.00	2063.00
Low_Noise_Accelerometer_Y_CAL	2.68	2.68	2.67	2.68
Low_Noise_Accelerometer_Z_RAW	1219.00	1218.00	1220.00	1221.00
Low_Noise_Accelerometer_Z_CAL	11.24	11.25	11.23	11.22
Wide_Range_Accelerometer_X_RAW	16.00	52.00	48.00	36.00
Wide_Range_Accelerometer_X_CAL	−0.63	−0.63	−0.65	−0.57
Wide_Range_Accelerometer_Y_RAW	−1060.00	−1056.00	−1088.00	−960.00
Wide_Range_Accelerometer_Y_CAL	−0.01	−0.03	−0.03	−0.02
Wide_Range_Accelerometer_Z_RAW	−17,352.00	−17,336.00	−17,352.00	−17,316.00
Wide_Range_Accelerometer_Z_CAL	10.38	10.37	10.38	10.36
Gyroscope_X_RAW	−24.00	−14.00	−20.00	−9.00
Gyroscope_X_CAL	−0.66	−1.19	−1.08	−0.81
Gyroscope_Y_RAW	43.00	78.00	71.00	53.00
Gyroscope_Y_CAL	0.37	0.21	0.31	0.14
Gyroscope_Z_RAW	−11.00	−8.00	−4.00	3.00
Gyroscope_Z_CAL	0.17	0.12	0.06	−0.05
Magnetometer_X_RAW	1023.00	1791.00	−2.00	1535.00
Magnetometer_X_CAL	14.58	20.73	13.82	17.27
Magnetometer_Y_RAW	9728.00	13,824.00	9216.00	11,520.00
Magnetometer_Y_CAL	−1.53	−2.69	0.00	−2.30
Magnetometer_Z_RAW	23,297.00	21,761.00	19,457.00	22,273.00
Magnetometer_Z_CAL	−34.93	−32.63	−29.17	−33.39
Pressure_RAW	20,384.00	20,385.00	20,385.00	20,384.00
Pressure_CAL	10,514.43	10,515.09	10,515.09	10,514.43
Temperature_RAW	32,862.00	32,862.00	32,862.00	32,862.00
Temperature_CAL	8.06	8.06	8.06	8.06
Axis_Angle_A_CAL	0.07	0.07	0.09	0.08
Axis_Angle_X_CAL	−0.14	−0.04	−0.02	0.14
Axis_Angle_Y_CAL	0.83	0.81	0.72	0.76
Axis_Angle_Z_CAL	−0.54	−0.58	−0.70	−0.63
Quaternion_0_CAL	1.00	1.00	1.00	1.00
Quaternion_1_CAL	0.00	0.00	0.00	0.01
Quaternion_2_CAL	0.03	0.03	0.03	0.03
Quaternion_3_CAL	−0.02	−0.02	−0.03	−0.02
